# A novel application of breadth first algorithm for achieving collision free memory mapping

**DOI:** 10.1371/journal.pone.0219490

**Published:** 2019-08-15

**Authors:** Saeed ur Rehman, Saeed Ehsan Awan, Fazel Rehman Mumtaz, Muhammad Asif Zahoor Raja

**Affiliations:** 1 Department of Electrical and Computer Engineering, COMSATS University Islamabad, Attock, Pakistan; 2 Kabul Depot, Kabul, Afghanistan; University of Zilina, SLOVAKIA

## Abstract

We are living in the world of handheld smart devices including smart phones, mini computers, tablets, net-books and others communication devices. The telecommunication standards used in these devices includes error correction codes which are integral part of current and future communication systems. To achieve the higher data rate applications, the turbo and Low Density Parity Check (LDPC) codes are decoded on parallel architecture which in turn raises the memory conflict issue. In order to get the good performance, the simultaneous access to the entire memory bank should be performed without any conflict. In this article we present breadth first technique applied on transportation modeling of the problem for solving the collision issue of Turbo decoders in order to get optimized architecture solution.

## Introduction

**Fig 1:**
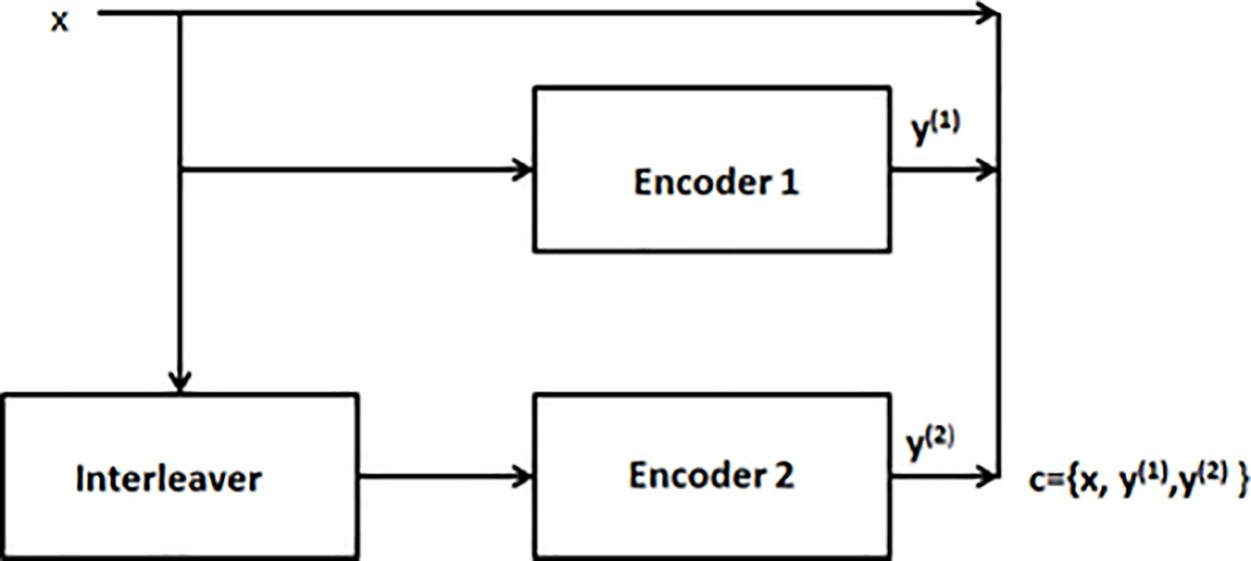
A generic turbo encoder.

The error correction codes are divided into two main categories, i.e., convolutional and block codes. Turbo codes belong to the class of convolutional codes. One advantage of convolution over block is the maximum likelihood soft decision decoding which is used to decode the data whereas block codes in contrast are hard decision decoded. Due to the best error correcting capabilities the turbo codes are also included in telecommunication standards such as Long Term Evolution (LTE) [1]. The soft decision decoding provides an advantage of reliability at each input data points and better performance as well. Soft decision decoding takes on a whole range of values in between where as hard decision decoding takes on a fixed set of possible values like zero and one [2]. A turbo encoder basically consists of an interleaver which is in between the two encoders as shown in Fig 1. The purpose of an interleaver is to arrange the data bits before they are being input to the 2nd encoder. The most critical part in the code design is choice of an interleaver. The two encoder output will be uncorrelated from each other because of the interleaver.

Turbo codes are based on two concepts. One is its concatenated coding and other is iterative decoding [3]. With much shorter component codes we can build a much more compound code. With concatenation the codes can be decoded much more easily. One of the main drawbacks of concatenation is error propagation [4]. If these errors are distributed to the separate code words, then the performance of the outer decoder can be enhanced. This aim can be achieved through an interleaver. Between the outer and inner encoders of concatenated codes an interleaver is placed. The decoded word is reapplied to the inner as well to the outer encoder and according to the need this process is repeated and thus forms the basics of iterative decoding.

Low Density Parity Check(LDPC) codes on the other hand are linear error correcting code in which the message is transmitted through a noisy channel. These codes are defined by parity check matrix H. It contains only a few number of non-zero entries [5]. Graphically they are represented by Bipartite graphs which helps to describe the decoding algorithm. These graphs give the complete representation of the code. For error detection they utilize the linear sum of information bits called parity bits [6]. The iterative decoding algorithm of LDPC codes shows the explicit parallelism and the capacity approaching performance shown by LDPC. These codes are included among the best error correcting codes [7] which are also part of telecommunication standards such as WiMAX (Worldwide Interoperability for Microwave Access) [8].

**Fig 2:**
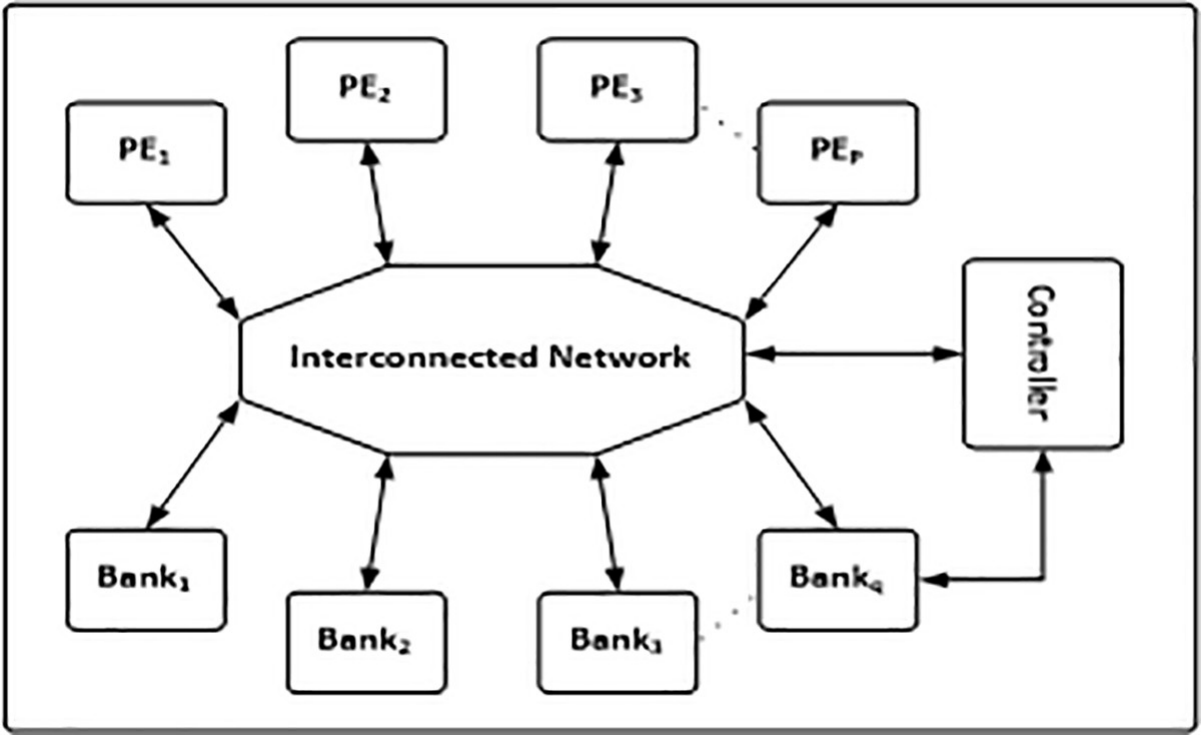
Parallel hardware architecture.

In order to get improved performance, the parallel processors in the hardware architecture is implemented which rises the memory conflict issue because several Processor Elements (PEs)process the same memory in the data bank at once. This parallel processors hardware architecture is shown in Fig 2. For both the Turbo and Low density parity check decodes the memory conflict problem is almost the same but the structure of decoder is different [9].

In order to achieve high performance of turbo codes, interleavers are used so the parity bits which are generated by two different encoders must be completely different. In order to increase the bandwidth, the interleavers are parallelized. Memory is divided into smaller memory banks however the memory conflict occurs because of the scrambling done by the interleaver. Generally, the data elements are accessed in natural order without any issue but the memory conflict arises when the data elements are retrieved in interleaved order. This issue increases the cost of system and reduces the system throughput [10–11].

In LDPC codes there is difference in code construction due to which the memory conflict issue in LDPC codes will be different from Turbo codes. In LDPC codes the memory conflict arises due to the irregular structure of H matrix, when various PEs try to access the same memory bank at the same time. This results in degradations in achievable throughput [12].

In this paper we propose a new technique of transportation modeling of the problem using breadth first technique for solving collision issue of Turbo decoders for obtaining optimized hardware architecture as a result. The proposed work optimizes the cost of network by using a targeted interconnection network for finding collision free solution to the problem.

The remainder of the paper consists of the related work, detail mapping of the problem, overview of the proposed work, presentation of the results with necessary interpretations and finally concluding remarks with some future recommendations.

## Related work

Turbo and LDPC codes are now most frequently used in the communication system because of their magnificent performance in error correction. These codes are the most crucial parts of the decoders as well. In order to solve the memory conflict issue that arises due to the parallel architecture, different approaches are proposed. An interleaver law is defined which normally maps the data elements in different memory banks so that they can be accessed at any time by the processing elements without any conflict.

### Interleaver law

An interleaver law is defined, which normally maps the data element in different memory banks, so that they can be accessed at any time by the processing elements without any conflict. If the interleaver law is well-defined, then this approach can reduce the cost of interconnected network. Such type of approach is proposed in [13].

### Run time approaches

In run time approaches, the conflicts are managed at run time. In these approaches the interconnected networks are used which increase the overall cost of the system. The throughput is also affected as the delay is introduced by conflict management mechanism and thus these approaches are not suitable for high data rate applications [14]. To find the conflict free memory mapping for the flexible LDPC decoders another approach based on simulated annealing algorithm is presented [15]. However, this approach fails to remove the computational complexity of the problem in which we cannot determine the complexity of the algorithm and the final architecture is not effective. To solve the memory mapping conflict issue in turbo decoders another approach called meta heuristic which is based on simulated annealing algorithm is presented [16–17]. These approach also failed to compute the complexity of the algorithm.

### Design time approaches

In literature design time approaches are proposed. [18] is a design time approach which is used to find collision free mapping for a target interconnected network. This approach can find the conflict free memory mapping with the targeted interconnection network. However, this approach is limited to partial turbo codes only. For the storage of banks information there are mapping matrices which store the information during the execution of this algorithm.

LDPC codes are very commonly used in communication system because they have error correction capabilities very close to the channel capacity. For LDPC codes MRMW (Memory Read Memory Write) mapping architecture is proposed in [19] to solve the memory conflict problem. In this architecture each data element has two mapping locations, one from where algorithm reads the data element and the second in which it writes the data element. But the resultant cost of the architecture is relatively high.

### Polynomial time approaches

The proposed previous approaches are heuristics therefore the computation time is not predictable. Polynomial time approaches have the solution for the memory conflict problem in which the conflict problem is solved in polynomial time. Thus the computational delay can be computed. An approach which solves the memory conflict issue for any type of codes in polynomial time is presented [20]. Its implementation shows a notable improvement in terms of computational time, complexity and cost as compared to state of art approaches which claims to overcome the approaches presented in [20–22]. The results from the experiments showed a great reduction in computational time to find the memory mapping for both the turbo and LDPC codes. In order to solve the memory conflict issue polynomial time memory mapping algorithm is embedded on on-chip to perform it at runtime [21].

In this work, we have presented a dedicated approach based on transportation problem modeling using breadth first algorithm in order to find a conflict free memory access to all the memory banks for every type of turbo codes. As we have discussed that conflict free memory mapping solutions are proposed in the literature. However, the final cost and execution time is needed to be optimized respecting the steering rule. Our aim is to optimize the final architecture to find the memory mapping which respects the targeted steering rule. In order to present our proposed work, first of all we will introduce the memory conflict problem in the next section.

## Parallel implementation of turbo codes and memory conflict problem

The parallel processor hardware architecture is implemented to get high throughput performance of codes which rises the memory conflict issue because several PEs try to access the same data bank at once. A typical parallel hardware architecture is shown in Fig 2.

To improve the error correction performance of turbo codes interleavers are used so the parity bits which are generated by two encoders must be completely different. In order to increase the bandwidth, the interleavers are parallelized. Memory is divided into smaller memory banks however the memory conflict occurs because of the scrambling done by the interleaver. The data elements are processed in natural order then the same data elements are processed from the memory banks in interleaved order. There exits memory conflict problem in such processing which increases the cost of system and reduces the system throughput.

The memory collision problem can be explained through an example of turbo codes. The code word is divided into four column (windows) in natural as well as interleaved order for the parallel processing. Every data element is accessed two times in turbo codes: first in the natural order and then in the interleaved order. One processing elements process each window.

### Problem formulation

**Fig 3:**
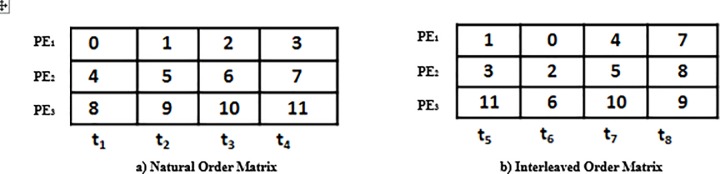
Data access matrices.

We have considered two matrices in order to explain the problem consider two matrices one for the natural order and the other for the interleaved order as shown in Fig 3. We have 12 data elements *L* = 12, three processing elements *P* = 3 and total 8 time instances *t* = 8.

### Problem of mapping

**Fig 4:**
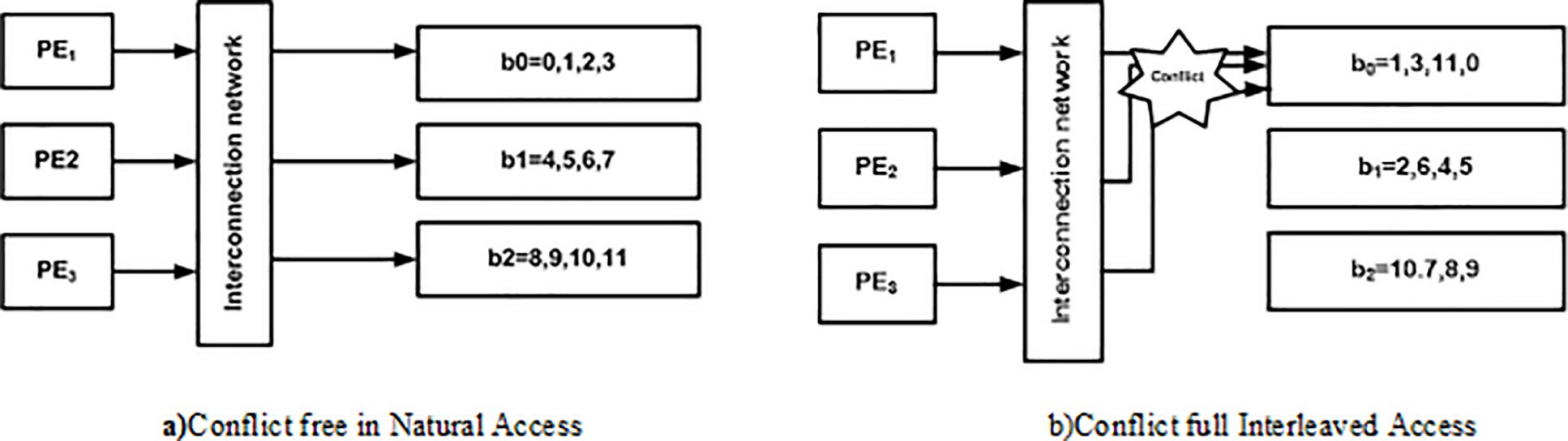
Memory collision problem in turbo codes.

There are three memory banks *B* = 3 so that each bank can be processed by *P* processing elements in both natural and interleaved order. As shown in Fig 3 the data elements are first processed by processing elements in natural order and then these data elements are accessed by processing elements in interleaved order. Due to this the memory conflict problem rises as shown in Fig 4 which increases the system’s latency in fetching the data element from the memory due to the existence of conflict management mechanism. This memory conflict problem also results in an increase in system’s cost and reduces the throughput of the system as well.

### Constraints

There are two constraints which need to be fulfilled in order to get a collision free memory mapping.

1. All the memory banks should be used once during each time instance.
2. One and only one memory bank is used to map each data element.

## Proposed work

In order to explain the proposed work, we will first explain the collision problem with the transportation problem techniques which can be applied to every example of turbo code to find collision free memory access. In this section we introduced the transportation modeling technique. However, the memory conflict problem for all the block lengths is not possible with transportation problem because there is limited exploration of possible memory conflict problem. Therefore, we have applied the breath first algorithm in order to solve the memory collision problem for all the block lengths. The breath first algorithm is able to keep track of all the possible solution of memory conflict problem.

We introduced few definitions associated to the bipartite graph in order to enlighten the approach proposed.

### Definitions

A graph *G*_*a*_ = (*V*_*n*_,*E*_*d*_) is a set of nodes *V*_*n*_, and a set of edges *E*_*d*_. If *v*,*w*∈*V*_*n*_ then (*v*,*w*)∈ *E*_*d*_ is incident to *v* and to *w* and *v* and *w* vertixes are adjacent.

A graph *G*_*b*_ = (*V*_*a*_*∪ V*_*b*_, *E*_*d*_) is bipartite which can divide the vertices set in *V*_*a*_, *V*_*b*_ in which every edge is incident to a vertex in *V*_*a*_, *V*_*b*_ such that *V*_*a*_*∩V*_*b*_ = {}.

The total edges indicate the degree of *v*. The degree regular graph has same degree for all vertexes while semi regular graphs have same degree either in *V*_*a*_ or *V*_*b*_. A two-matching (2 matching) *M* of *G*_*b*_ = (*V*_*a*_*∪ V*_*b*_, *E*_*d*_) consist of subset of *E*_*d*_ in which every node in *G*_*b*_ is inclined on maximum two edges from *M*. This two matching *M* can be known as two factor (2 factor) if all nodes are inclined with fixed number of two edges of *M* [23].

For a two factor, [24] proposed two theorems:

Theorem#1: Graph (*2y*-regular) consists of two factor, where *y* is integer.

Theorem#2: Graph (2*y* + 1 regular) consist of two factor.

In [24], these theorems concluded two corollaries;

Corollary#1: Graph (2*y*-regular) consists of *y* two factor.

Corollary#2: Graph (2*y* + 1 regular) consist of *y* two factor with one one-factor.

Definition 1: In semi regular bipartite graph *G*_*b*_ *= (V*_*a*_ *∪ V*_*b*_, *E*_*d*_*)*, vertices in *V*_*a*_ or *V*_*b*_ are of the same degrees. A semi two factor is a two regular sub-graph in *G*_*b*_ having *2K* vertices and all nodes are inclined to fixed two edges and *K* = Min(|*V*_*a*_|, |*V*_*b*_|).

Definition 2: Turbo-Bipartite-Graph T.B.G will be bipartite = (*T∪ L*, *E*_*d*_) such that the time instances are represented by *T* and *L* represent the set of data element vertexes. An edge *(t*, *l)* is incident to the data element vertex *l* and to the time instance vertex *t* if *l* needs to be processed at *t*.

The T.B.G has two distinct properties: -

Property#1: The number of accesses of processing elements to the data elements at a particular time instance is always equal i.e., the number of processing elements is equal to the number of banks, which shows that the bipartite graph is always semi regular so All the time nodes have equal degree (*d*_*t*_ *= P = B*).

Property#2: Every data node in T.B.G have equal degree. We have to access all the data elements exactly two times first in natural order then in interleaved order so its degree will be *d*_*d*_ *=* 2.

Here we can introduce a corollary given as:

Corollary#3: A T.B.G having *d*_*t*_ *= 2y* or 2*y*+1 have y semi two factors. As it is semi regular graph on both vertices with *d*_*d*_ *= 2* we will always find semi two factor in step 2 in case of Turbo Bipartite Graph.

All the two factor consist of cycles for the graph vertexes from cycle 1 (*2K* vertexes) to *K* cycles (2 vertexes).

**Fig 5:**
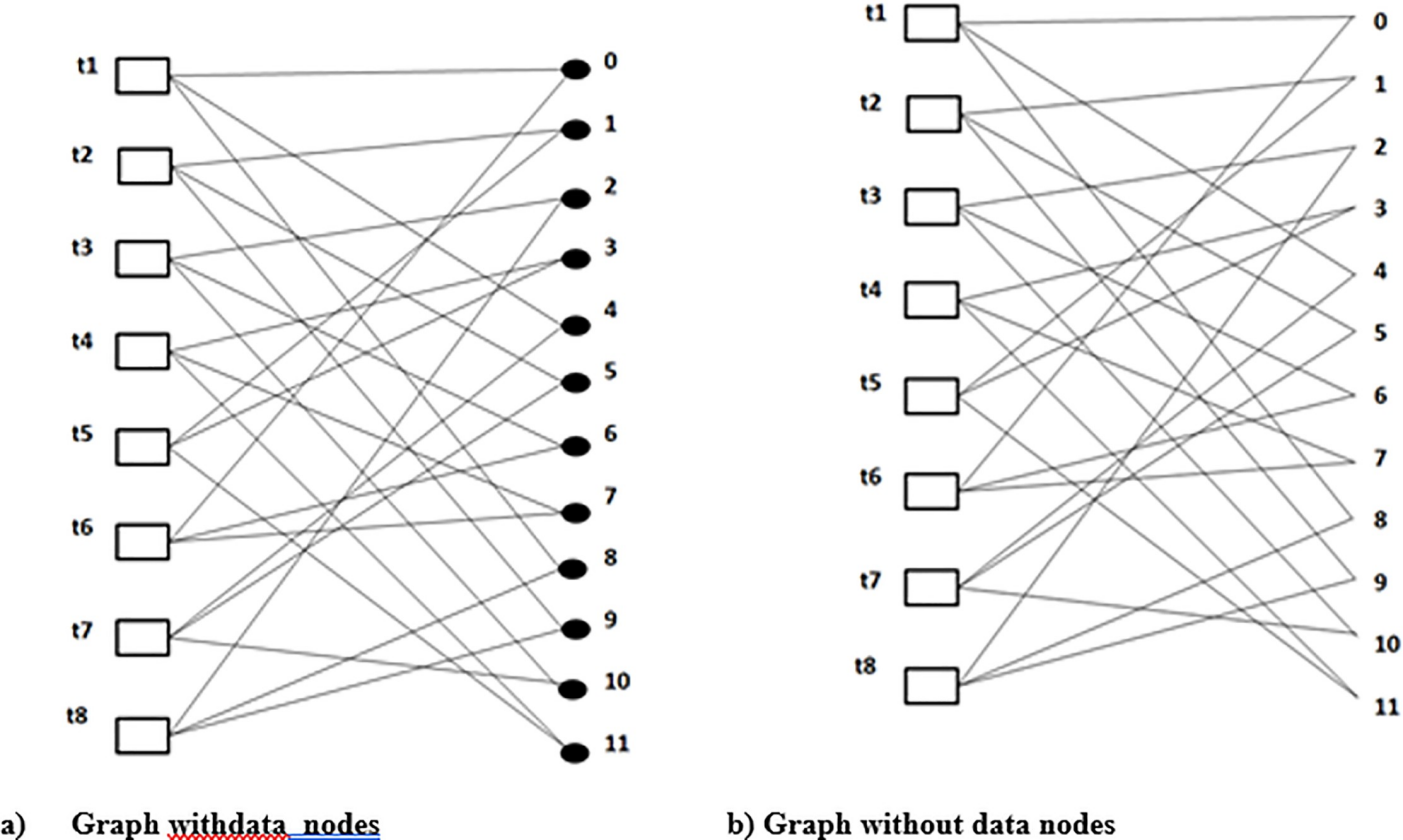
Turbo bipartite graph.

Two *u* can be allotted to edges of all the even cycles which represent that every data node in a semi two factor of T.B.G is assigned to two colours which can result in two memory banks. The proposed work is based on three steps:

Step 1: A T.B.G is formed using the natural and interleaved data order (as shown in Fig 5)

Step 2: We split the T.B.G into *Y* semi 2-factors with the use of approach whose modeling is based on transportation problem [24].

Step#3: Apply the breadth first algorithm for *Y* semi 2-factors to obtain the conflict free memory mapping.

In the first step, T.B.G is formed using the natural and interleaved data order. Join the two connected edges along one another data node as shown in Fig 5(A). Then take away all the data nodes in order to create a graph which is regular(*G*_*1*_) as shown in Fig 5(B). Every two factor consist of cycles which cover all of the vertices in graph from 1 cycle of *2K* vertixes to *K* cycles *2K* vertixes. Moreover, a particular cycle *c*_*i*_ (where *i = 1* in cycle 1 i-e *c*_*1*_ *and so on*) consist of even edges which shows even time nodes as *c*_*i*_ cycle in *G*_*1*_ is of even number due to the division of vertex set *T* into natural and interleaved order. Two colours can be assigned to every even cycle such that edges in cycle *c*_*i*_ including all the two factor in *G*_*1*_ are of two colours.

There are three steps in which the transportation problem is modeled [24].

- Network Model
- Matrix Model
- Applying Algorithm

**Fig 6:**
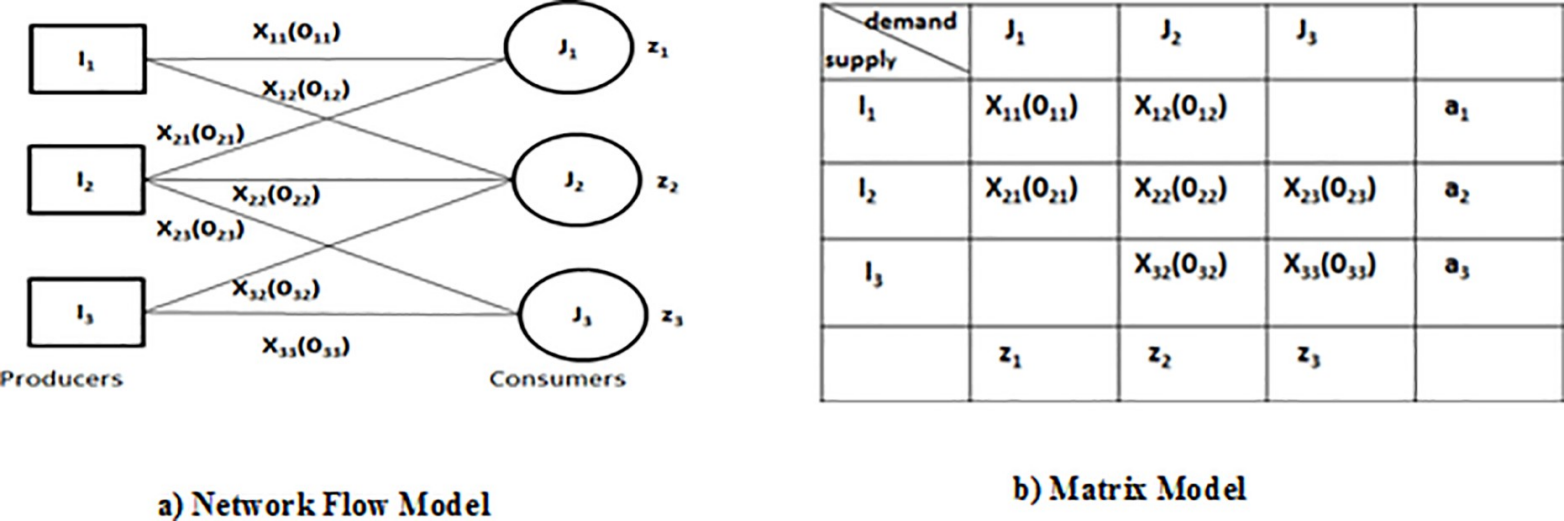
Transportation problem.

In order to explain transportation problem algorithm, we have presented a graph which is bipartite [24] in network model shown in Fig 6(A). All the producers are represented by a first node whereas all the consumers are represented by a second node.

Consider we have the set of producers I such that *i*^*th*^ producer will have to supply an item of units *a*_*i*_. We have the set of consumers J, and the *j*^*th*^ consumer will need *z*_*j*_ units of that item. Consider *a*_*i*_, *z*_*j*_ is greater than zero. Let *o*_*ij*_ is the cost of transportation of one item on *l*_*ij*_ where *l*_*ij*_ is the link of producer *i* towards consumer *j* (*l*_*ij*_) and *x*_*ij*_ is the total capacity of that particular link (*l*_*ij*_) in which the maximum number of items can be shipped (See Fig 6A). The transportation problem algorithm is modeled as a matrix in the mentioned matrix model Fig 6B. All the producers are represented by rows and all the consumers are represented by columns. *X*_*ij*_ and *0*_*ij*_ represents the corresponding capacity and cost of route *I*_*ij*_.

We will apply the transportation problem modeling to the example shown in Fig 3. Turbo bipartite graph is constructed in the first step as shown in Fig 5 where the set of vertexes *t* gives all of the time nodes and *d* gives all the data element nodes. In order to explain the transportation problem modeling clearly some definitions theorems and corollaries are stated above.

**Fig 7:**
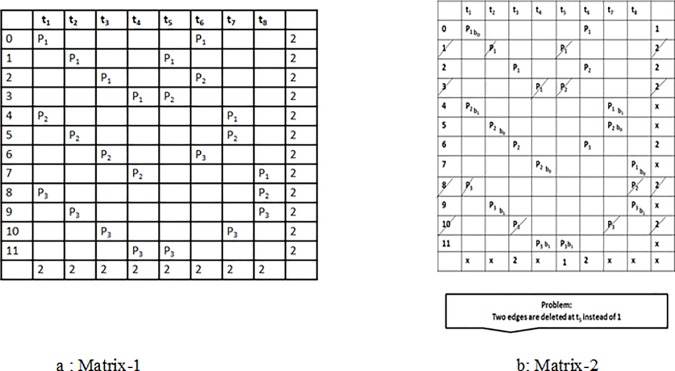
Transportation matrix.

The second step of transportation modeling is generation of transportation matrix as shown in Fig 7A. Every data node is taken as producers and every time nodes as considered as consumers. We have the processors at a given time instances in the matrix model of transportation technique. We have assigned the processors at a given time instances in the matrix model of transportation technique. The cost and capacity of the route are considered as 1 given as *1(1)* in the matrix. As given in example each data element is accessed in natural order and the in the interleaved order. Each consumer’s demand and each producer’s supply is kept as 2 for finding the semi-2 factors. Since every route has the equal cost and capacity so we did not write *1(1)* with each processor in the matrix.

The algorithm starts by constructing a cycle *c*_*1*_ from the first route *I*_*ij*_ with consumer *t*_*1*_. After the assignment of bank to the route the demand and supply of *t*_*1*_ and *d*_*1*_ is reduced to 1. The same bank is assignment to the other route. After that the algorithm selects the next route *I*_*k*_ connected with *t*_*1*_. After the bank assignment of both route the demand of *t*_*1*_is completed so all other routes connected with *t*_*1*_ are removed according to semi 2 factors. Now the algorithm selects the next route *I*_*k*_ and assign it a memory bank to fulfill the consumer’s demand. Algorithm keeps on repeating the same process selects the route and assign them banks, providing producer and demand of the consumer is decreasing sideways. We should not have any producer with supply of 1 and have no consumer with a demand of 1 at the end of cycle *c*_*1*_.

The algorithm tells at this point if any consumers are left with unfulfilled demand the algorithm constructs another cycle *c*_*2*_ to fulfill the demand of left consumers. According to targeted steering rule (barrel shifter) the algorithm selects the route and assigns the memory banks.

After this the algorithm starts constructing the cycle *c*_*2*_ as done in case of cycle *c*_*1*_and assigns memory banks to all the routes. If all the consumer’s demands are fulfilled, the approach tests if all the semi-2 factors are found, if yes, then the approach is completed if no, the algorithm starts constructing the cycle again as explained earlier.

**Fig 8:**
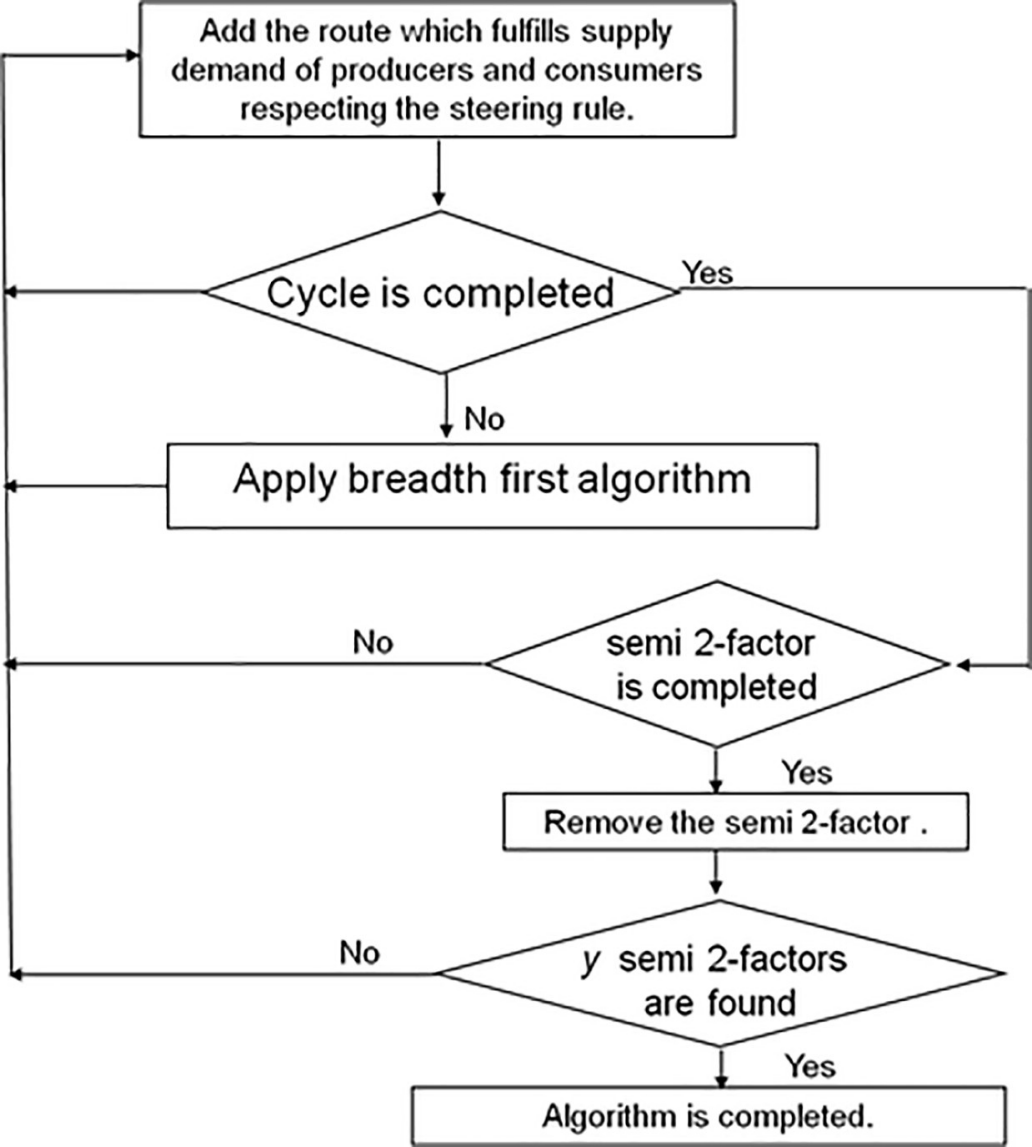
Partitioning algorithm.

We applied the above mentioned algorithm in our example. It can be seen in the Fig 7B, that this is not a right solution for finding the cycle as two edges are deleted at t_5_ instead of 1 using the process shown in Fig 8. The cycle is not completed at the selected node and no other cycles are recorded so we cannot find conflict free memory mapping. In order to overcome this problem, we have used breath first algorithm for memory mapping which is based on transportation problem modeling. In this approach, the cycle is initiated from a node and every node is recorded at that particular step only one of the nodes is selected by the algorithm and we complete the cycle by using that particular selected node. Moreover, in case of deletion of the additional rows or when the cycle is not completed, other nodes are searched one by one in the form of tree. The whole process is based on breadth first algorithm approach for the construction of a cycle till the completion of the cycle. The first step is the construction of turbo bipartite graph shown in Fig 5. The second step of transportation problem modeling involves a transportation matrix shown in Fig 7A above.

In step 3, Breadth first search algorithm is used for searching a graph or data structures. It starts from some irrational node of a graph or table which is referred as the search key and it first checks all the neighbor nodes and then moves to the next level of searching the other levels of nodes. It can solve any problem the functionality is enhanced because of the breadth first search algorithm.

In Breadth first algorithm the cycle basically starts from a node and all the other nodes connected with that selected node are recorded. The algorithm selects the first route which is at *I(0*,*1)* and assign it a memory bank *b*_*0*_. The next node connected with *t*_*1*_ are at *I (4*,*1)* and *I (8*,*1)*. The next route we selected is *I (8*,*1)* and assign it memory bank b_1_, the other route of *d*_*8*_ i.e., *I(8*,*8)* is also assigned the same bank *b*_*1*_. Now the demand of *t*_*1*_ is fulfilled so the other node connected with *t*_*1*_ i.e., *d*_*4*_ is deleted. Now we are at point *I(8*,*8)* the two other nodes connected with *t*_*8*_ are at point *I(7*,*8)* and *I(9*,*8)*, we selected *I(9*,*8)* and assign it memory bank b0 the other route *I(9*,*2)* is also assigned bank *b*_*0*_. Now we are at point *I(9*,*2)* two nodes are connected one at *I(5*,*2)* and the other at *I(1*,*2)*, we selected *I(5*,*2)* and assign memory bank *b*_*1*_ to it, now both demands of *t*_*2*_ are fulfilled so delete the other data element connected with *t*_*2*_ i.e., *d*_*1*_so it got deleted. The other route of *I(5*,*2)* i.e., *I(5*,*7)* is also assigned same bank *b*_*1*_. At node *I(5*,*7)*, we have two other nodes one at *I(4*,*7)* and *I(10*,*7)* we selected (10,7) and assign it bank *b*_*0*_, now the demand of *t*_*7*_is fulfilled so other node connected with *t*_*7*_ i.e., *d*_*4*_ is deleted. The other route *I(10*,*3)* is also assigned bank *b*_*0*_. The other two nodes connected with *I(10*,*3)* are *I(6*,*3)* and *I(2*,*3)* we selected *I(6*,*3)* and assign it memory bank *b*_*1*_ now both the demands of t_3_ are fulfilled so the node at *t*_*3*_ i.e., data element *d*_*2*_is deleted. The other route of *I(6*,*3)* which is at *I(6*,*6)* is also assigned the same bank *b*_*1*_. Now at node *I(6*,*6)*, we have two other nodes connected with it i.e., *I(7*,*6)* and *I(0*,*6)*, we selected *I(0*,*6)* to assign the bank *b*_*0*_ in order to complete the cycle *c*_*1*_, thus the other node at *I(7*,*6)* i.e., data element *d*_*7*_ is deleted as both the demands of *t*_*6*_ is fulfilled.

**Fig 9:**
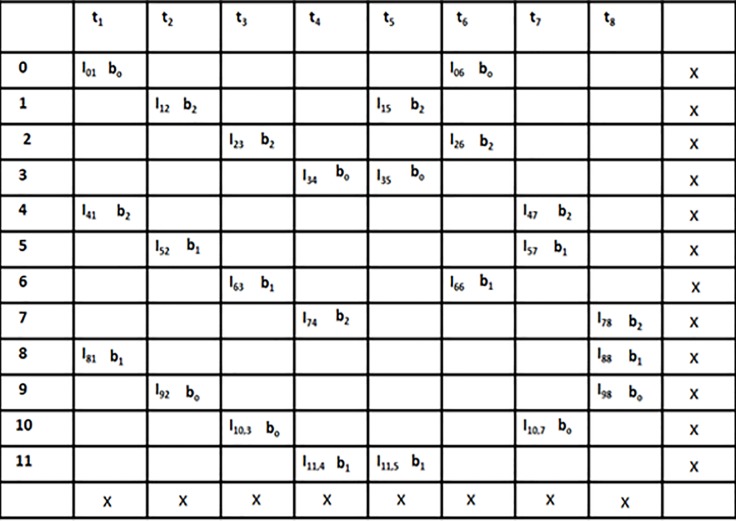
Complete mapping matrix.

By the end of the cycle *c*_*1*_, two data elements *d*_*3*_ and *d*_*11*_ are left to be assigned with memory banks, so we constructed the cycle *c*_*2*_to complete the assigning process. Cycle *c*_*2*_ is started from the data element *d*_*3*_ and the route 1st route *I(3*,*4)* is assigned with bank *b*_*0*_, the two other nodes at *I(3*,*4)* are *I(7*,*4)* and *I(11*,*4)*. As *I(7*,*4)* is already deleted so we selected the next node at *I(11*,*4)* and assigned it with bank b_1_, the other route i.e., *I(11*,*5)* is also assigned with the same bank b_1_. Now we are at point *I(11*,*5)* we selected the only left route i.e., *I(3*,*5)* and assign it with bank *b*_*0*_ in order to complete the cycle *c*_*2*_. At the end of cycle *c*_*2*_ all the deleted routes i.e., 1,2, 4 and 7 are assigned bank *b*_*2*_ to complete the final mapping. The complete mapping is shown in Fig 9 below:

## Results and discussion

The proposed approach is applied to the standard of 3GPP LTE turbo decoders. In order to increase the throughput, parallel architecture is used. Unfortunately, the parallel architecture has memory conflict problem in the implementation of turbo decoder. We have applied our proposed approach on the above mentioned standards which can solve its memory conflicts issues for obtaining optimized results. These results demonstrate the effectiveness of the proposed method. In this section we presented the results which we obtained in our experiments in terms of CPU time and the area. In order to calculate area in these experiments 90nm technology is used from STMicroelectronics. The area is given in NAND gate identical of STMicroelectronics. In our experiments we compared the CPU time of our proposed approach with existing approaches using Dell PC i7 core 2.67GHZ.

### Experiment 1

In the first set of experiments, we have compared the transportation architectural area for different parallelism (*P* = 4,8,16) using fixed block length with existing approaches. We have considered the following two test cases.

#### Case 1

In the 1st case we have performed the experiment for calculating the area for different values of parallelism (4,8,16) and fixed block length *L* = 5120 and compared the results with existing approaches [16] and [18].

The resultant area compared with Benedetho approach [16] and SAGE approach [18] along with *L* = 5120 and different values of *P* = 4, 8, 16 is shown in Table 1. The total area is same so the cost is same for both [16], [18] and the proposed approach.

**Table 1:** Resultant area of turbo decoder for different parallelism (*L* = 5120).

Parallelism	Total Area(Eq Nand gate) nm
**4**	2.938880*10^6^
**8**	2.938880*10^6^
**16**	2.938880*10^6^

#### Case 2

In the 2nd case we have performed the experiment for different values of parallelism (4,8,16) and fixed block length *L* = 4480 and compared the results with the existing approaches. The resultant area compared with [16], [18] along with L = 4396 and different values of *P* = 4, 8, 16 is shown in Table 2. The total area is almost same so the cost is same for both [16], [18] and the transportation.

**Table 2:** Resultant area of turbo decoder for different parallelism (*L* = 4480).

Parallelism	Total Area(Eq Nand gate) nm
**4**	2.571520*10^6^
**8**	2.571520*10^6^
**16**	2.571520*10^6^

### Experiment 2

In this set of experiments, we have compared the transportation architectural time in *ms* for different values of parallelism and different block lengths with existing approaches.

#### Case 1

The CPU time in milliseconds for the existing approaches [16] [18] and the proposed approach is shown in Table 3. The Table indicates there is a huge time difference between our proposed approach and the existing approaches.

**Table 3:** CPU time for various mapping approaches for L = 5120.

Parallelism	Transportation	[16]	[18]
P = 4	500	3500	3000
P = 8	490	3500	5000
P = 16	490	24200	5000

**Fig 10:**
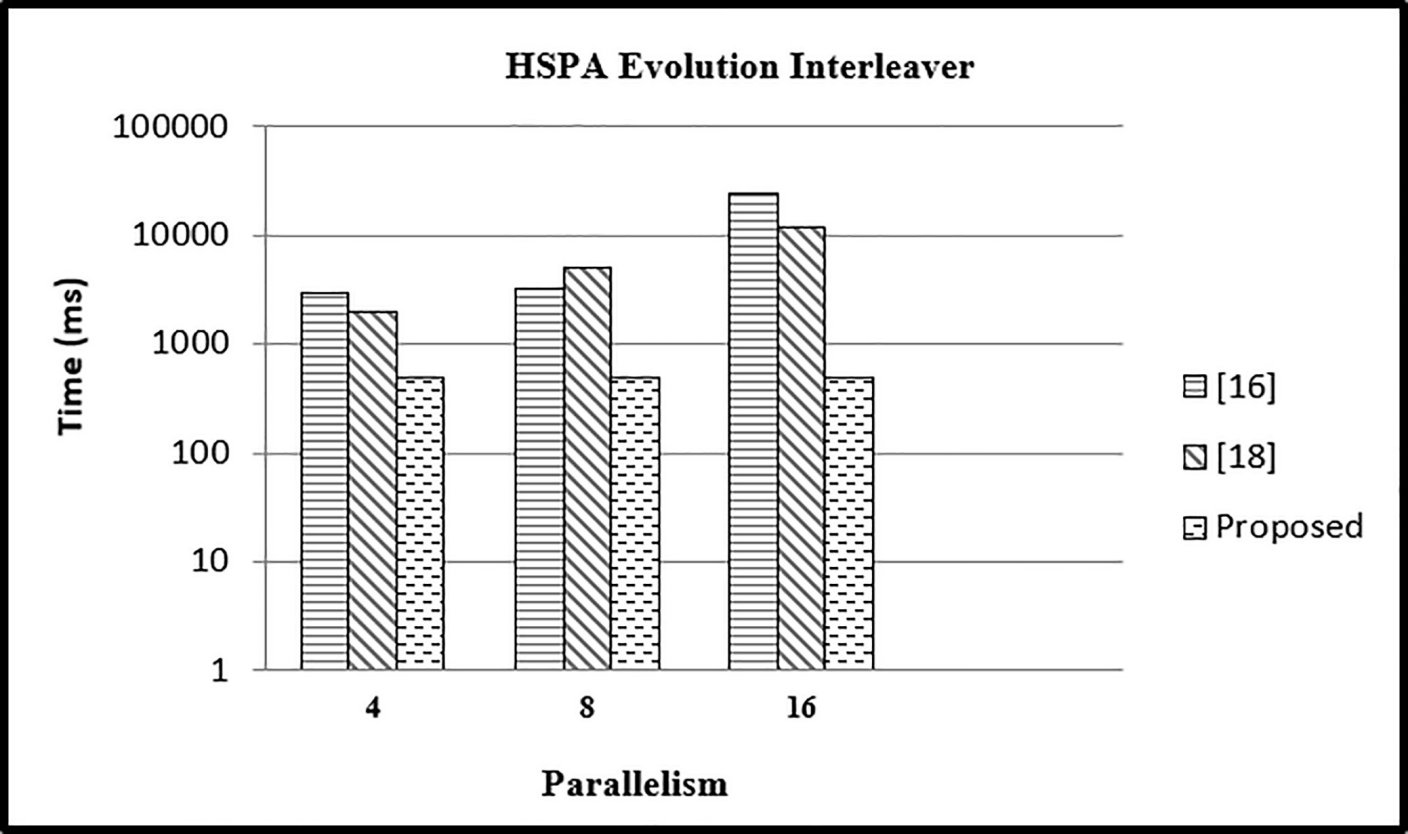
Comparison of time for different memory approaches.

With an increase in parallelism the time almost remains constant while in [16] [18] it is increasing with an increase in parallelism. Therefore, the polynomial time algorithm provides a great advantage in terms of time as compared to [16] and [18] as shown in Fig 10.

#### Case 2

In the 2nd case we have performed the experiment for different values of parallelism (16,32,64) and fixed block length *L* = 4480 and compared the results with existing approaches.

**Fig 11:**
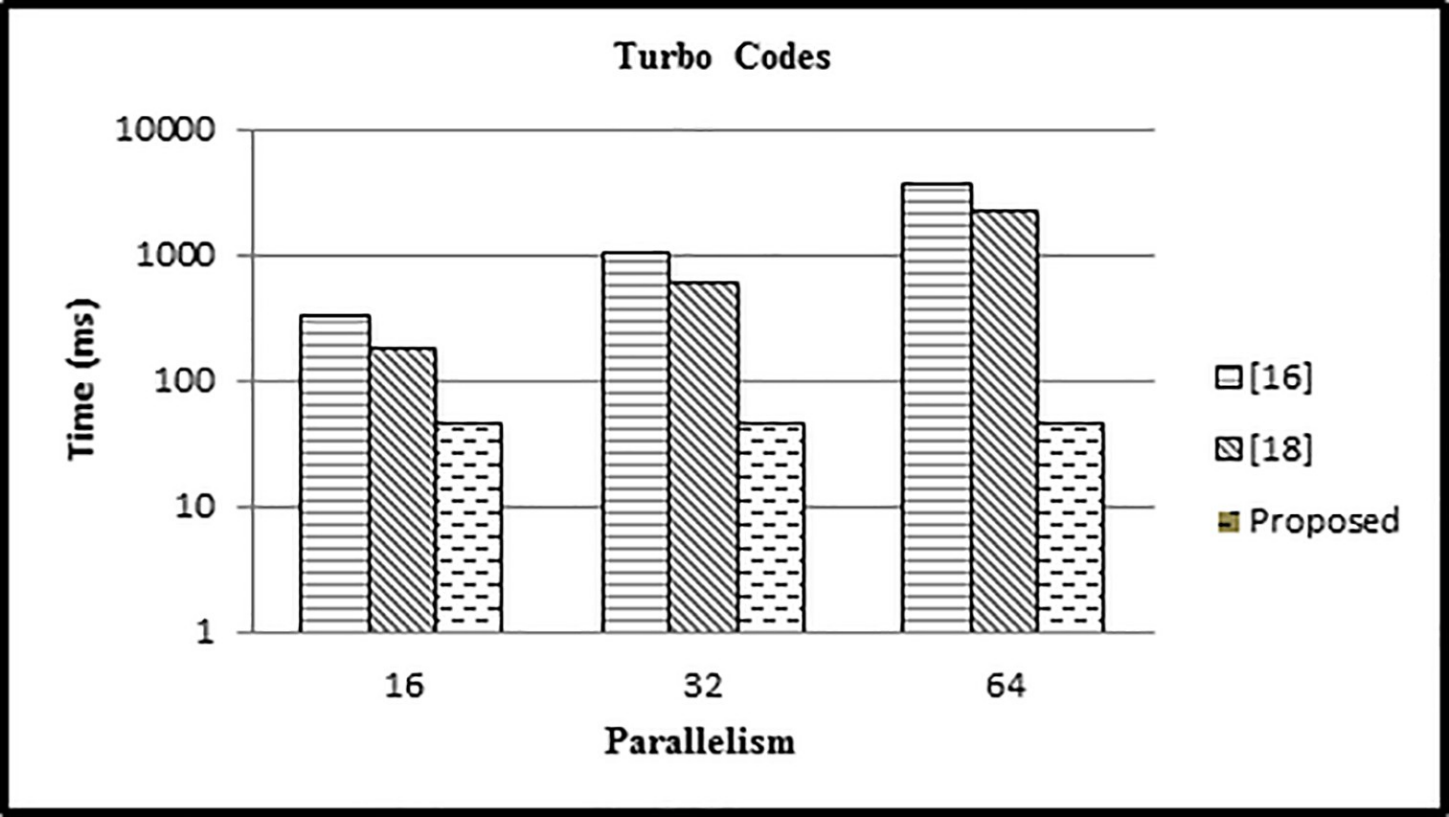
Comparison of time for various mapping approaches (L = 4480).

In case 2, the value of *L* is kept constant as: *L* = 4480, with varying values of P = 16,32,64. It can be seen in Fig 11 that the time in our approach is reduced as compared to other two approaches.

#### Case 3

**Fig 12:**
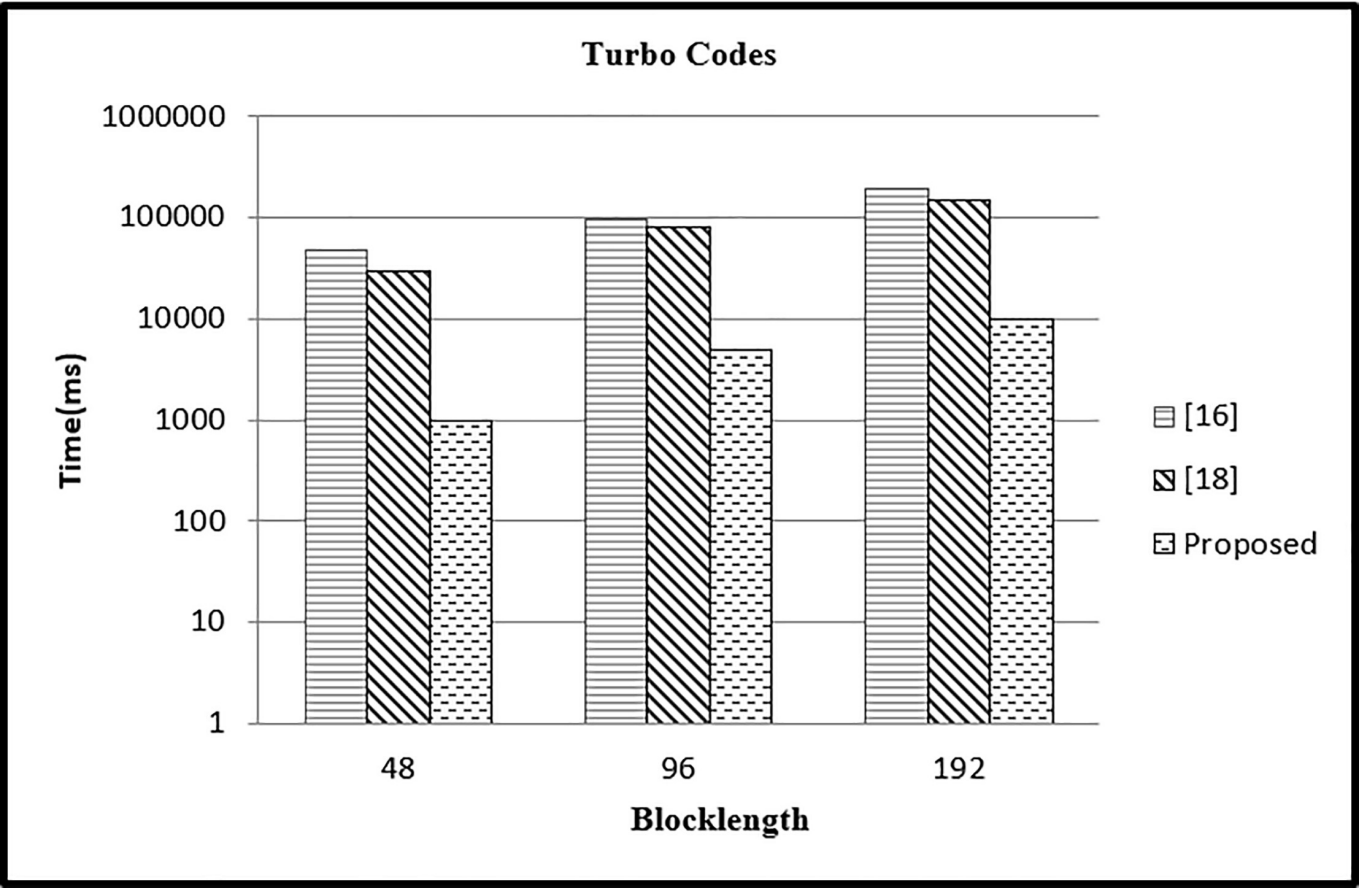
Comparison of time with other mapping approaches (*P* = 4).

In third case, we have performed the experiment for different block lengths (48,96,192) and fixed parallelism *P* = 4 and compared the results with existing approaches. In case 3, the value of *P* is kept constant as *P* = 4 with varying values of *L* = 48,96,192. It can be seen in Fig 12, that the time in our approach is reduced as compared to other two approaches.

#### Case 4

**Fig 13:**
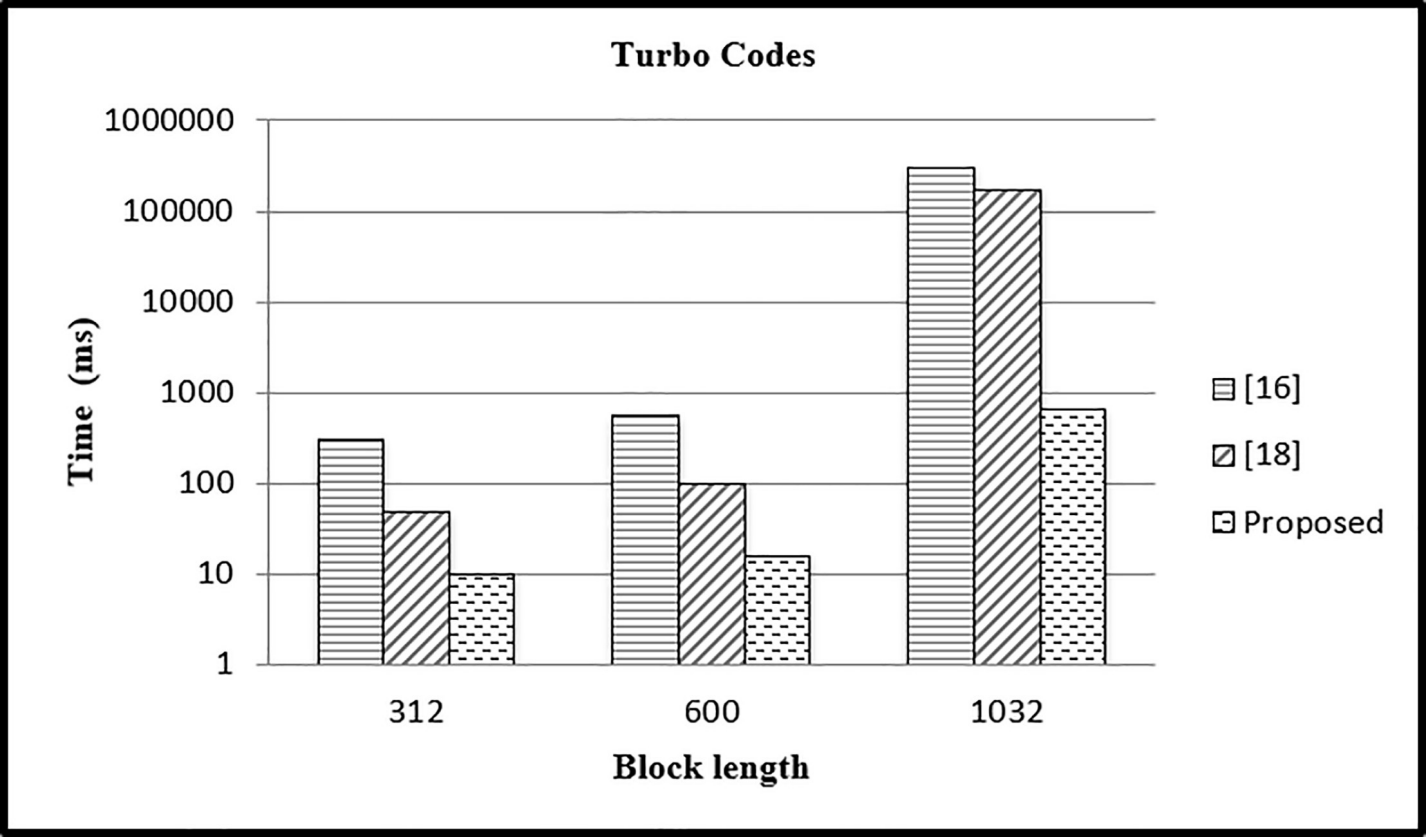
Comparison of time with other mapping approaches (P = 8).

In this case, we have performed the experiment for different block lengths (312, 600,1032) and fixed parallelism *P* = 8 and compared the results with existing approaches. In case 4, the CPU time is calculated for *P* = 8 and different values of *L* = 312,600, 1032 and results are presented in Fig 13. The CPU time in our case is reduced as compared to other two approaches.

#### Case 5

**Fig 14:**
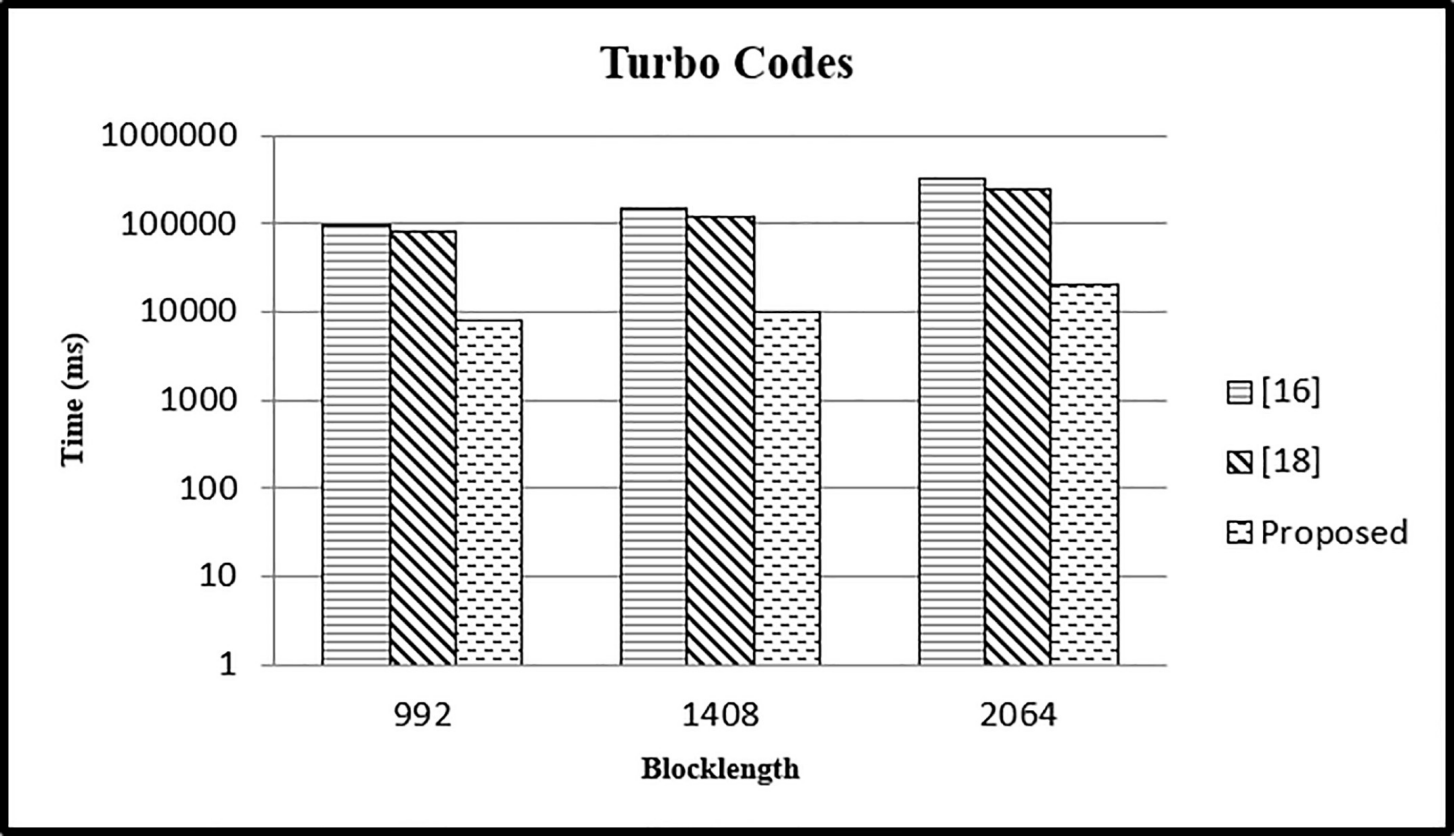
Comparison of time with other mapping approaches (*P* = 16).

In this case we have performed the experiment for different block lengths (992,1408,2064) and fixed parallelism *P* = 16 and compared the results with existing approaches. In case 5 for varying values of *L* = 992,1408,2064 and fixed value of *P* = 16 the CPU time is calculated. It is clear from Fig 14 that the time in our case is reduced as compared to other two approaches.

In this section, the results of simulations are presented for memory conflict problem based on transportation modeling using the breadth first algorithm. The proposed scheme is applied for number of scenarios and compared the results with existing approaches in terms of the CPU time and Area. Total area is same for varying values of *P* and for *L* = 5120 and *L* = 4480 in both [16, 18] and our proposed approach as shown in Table 1 and in Table 2. The cost is also same but the processing time is reduced in all the cases. Thus the polynomial time gives advantage in reducing the computational time in order to find a memory mapping free of collision for optimizing the resultant architecture. The results shown in this section is based on Barrel shifter as targeted network. This work can be extended to show results based on other networks exploration such as Butterfly and Benes networks.

## Conclusions

In this paper, we have presented the breadth first algorithm technique applied on the transportation problem modeling for solving the conflict issue of all types of turbo codes for designing parallel processor decoder architecture. This approach provides concurrent access to memory banks by finding collision free mapping. The proposed approach in implemented for different test cases for achieving collision free mapping by successful execution of the algorithm. As a result, the final architecture is optimized.

In future, we can explore the depth first algorithm for this problem instead of breadth first algorithm in order to get more optimized. Furthermore, the interconnection networks of the architecture can also be explored. Different interconnection networks like BENES and Butterfly networks can be explored. This may further optimize the resultant architecture in order to get better throughput performances.
